# Eudragit^®^ L100/*N*-Trimethylchitosan Chloride Microspheres for Oral Insulin Delivery

**DOI:** 10.3390/molecules18066734

**Published:** 2013-06-07

**Authors:** Etienne Marais, Josias Hamman, Lissinda du Plessis, Righard Lemmer, Jan Steenekamp

**Affiliations:** 1 Department of Pharmaceutics, North-West University, Private Bag X6001, Potchefstroom 2520, South Africa; E-Mails: 20402317@nwu.ac.za (E.M.); 119483388@nwu.ac.za (L.P.); 2 Centre of Excellence for Pharmaceutical Sciences, North-West University, Private Bag X6001, Potchefstroom 2520, South Africa; E-Mails: sias.hamman@nwu.ac.za (J.H.); 12932574@nwu.ac.za (R.L.)

**Keywords:** Eudragit^®^ L100, insulin, microspheres, *N*-trimethylchitosan chloride, oral peptide delivery, paracellular transport

## Abstract

Effective oral delivery of protein and peptide drugs remains an active topic in scientific research. In this study, matrix type microspheres were prepared with Eudragit^®^ L100 containing *N*-trimethylchitosan chloride to improve the permeation of insulin across the intestinal epithelium via the paracellular pathway. Insulin loaded microspheres were initially formulated in accordance with a factorial design (2^3^) and manufactured by means of a single water-in-oil emulsification/evaporation method. Based on external and internal morphology two microsphere formulations were selected from the initial formulations for further investigation in terms of particle size, dissolution behaviour and *in vitro* insulin transport across excised rat intestinal tissue. The initial eight microsphere formulations exhibited drug loading capacities ranging from 27.9–52.4% with different shapes and internal structures. The two selected microsphere formulations had average particle sizes of 157.3 ± 31.74 µm and 135.7 ± 41.05 µm, respectively, and mean dissolution time values for insulin release of 34.47 and 42.63 min, respectively. *In vitro* transport of insulin across excised rat intestinal tissue from the two selected microsphere formulations was 10.67–fold and 9.68–fold higher than the control group (insulin alone). The microsphere delivery system prepared from Eudragit^®^ L100 containing *N*-trimethylchitosan chloride is therefore a promising candidate for effective oral insulin delivery.

## 1. Introduction

The oral route is in general the most widely and preferred route of drug administration because it improves patient compliance. This is due to the fact that oral administration avoids the pain and discomfort as well as the possibility of infections associated with injections. However, oral administration of peptide and protein drugs is generally associated with poor bioavailability (typically less than 2%) [1]. The poor bioavailability of these drugs can be attributed to their large molecular size, hydrophilicity and their susceptibility to enzymatic degradation [2,3]. The physiology of the gastrointestinal tract also contributes to their low bioavailability due to various physical and biochemical barriers such as the physical barrier of the lipid-bilayer membranes of the intestinal epithelia, enzymatic degradation and active efflux transporter systems [4].

Due to advances in biotechnology, chemistry and molecular biology the production of large quantities of structurally diverse peptides and proteins is now a common activity. These developments have increased the need for novel delivery systems for peptide and protein therapeutic agents [1]. To overcome the obstacles associated with oral peptide and protein drug delivery, various strategies have been suggested, such as chemical modifications including pro-drug strategies, structural modifications such as PEGylation and lipidization; targeting of transporters or tissues such as receptor-mediated endocytosis and gut associated lymphoid tissue (GALT); and formulation technologies such as particulate carriers, inclusion of absorption enhancers in dosage forms and mucoadhesive/bioadhesive systems [5].

Promising results have been obtained with combinations of some of these strategies, for instance, Ziv *et al.* [6] demonstrated that a combination of a bile acid (*i.e.*, sodium cholate) as permeation enhancer and aprotinin as a proteinase inhibitor was more effective in improving the oral bioavailability of insulin as well as pancreatic RNase than either of the agents alone. Hosny *et al.* [7] demonstrated that the inclusion of sodium salicylate as an absorption enhancer into enteric–coated insulin-containing capsule formulations resulted in a 25–30% reduction in plasma glucose levels when inserted in the stomach of streptozotocin-induced diabetic rats.

In terms of formulation strategies, microparticles as a dosage form provide benefits such as rapid emptying from the stomach as well as more reproducible transit through the small intestine and colon. Their increased surface area facilitates rapid drug release and more reproducible absorption than conventional dosage forms [8]. Further advantages of microsphere drug delivery systems include effective protection of encapsulated drugs against degradation, increased drug solubility, reduced adverse or toxic effects, site-specific drug delivery and controlled drug release [9].

The surface area of the paracellular drug transport pathway (*i.e.*, drug absorption through the intercellular spaces between epithelial cells) in the gastrointestinal tract is estimated to be about 200 to 2,000 cm^2^. Therefore, this pathway should not be underestimated for effective peptide and protein drug delivery. Furthermore, even very small quantities (pM–nM range) of potent peptide drugs may be sufficient to produce the required therapeutic effect [10]. Modulation of the tight junctions between adjacent epithelial cells can be achieved by absorption enhancers such as chitosan and its derivatives (e.g., *N*-trimethylchitosan chloride or TMC) [11].

Chitosan [(1→4)-2-amino-2-deoxy-β-d-glucan] is a polysaccharide comprised of co-polymers of glucosamine and *N*-acetylglucosamine [12]. It has been shown that chitosan is capable of improving the paracellular transport of hydrophilic macromolecular drugs, however, chitosan is only soluble in acidic environments. Therefore, chitosan and its salts may not be a suitable absorption enhancer for targeted delivery to specific sites of the intestine such as the jejunum or ileum [12,13]. In fact, Kotzé *et al.* [14] proved that chitosan hydrochloride and chitosan glutamate were unable to cause a reduction in transepithelial electrical resistance (TEER) of Caco-2 cell monolayers at a pH of 7.4—which is an indicator of the opening of tight junctions—and they were also unable to cause an increase in the transport of [^14^C]-mannitol. This was attributed to the insolubility of these two salts at this neutral pH. These authors concluded that there is a need for chitosan derivatives with an increased solubility, especially at neutral and alkaline pH values. To overcome the solubility issues of chitosan, *N*-trimethylchitosan chloride (TMC), was synthesized [15]. TMC is a quaternized chitosan derivative that can be synthesized with different degrees of trimethylation [16]. Like chitosan, TMC possesses bioadhesive properties and acts as a paracellular absorption enhancer, however, in comparison to chitosan, TMC exhibits solubility in neutral and alkaline environments [11,17]. Kotzé *et al.* [18] demonstrated that TMC with a high degree of quaternisation was able to cause a 35% decrease in the TEER of Caco–2 cell monolayers at pH 7.4. Transport studies with [^14^C]-mannitol gave results that were in agreement with the TEER reduction and increases between 31 to 48-fold at a concentration range of 0.05–1.5% w/v of the TMC were obtained. In another study by Hamman *et al.* [19], it was found that the maximum reduction in TEER (47.3 ± 6.0% at a concentration of 0.5% w/v and pH 7.4) was reached with TMC with a degree of quaternisation of 48%, and this effect did not increase further with higher degrees of quaternisation. In agreement with the TEER results, the transport of the model compounds ([^14^C]-mannitol and [^14^C] PEG 4000) increased with an increase in the degree of quaternisation of TMC reaching a maximum at 48%. Futhermore, chitosan and its derivatives are generally considered safe, biodegradable and non-toxic [11]. 

The aim of this study was to combine the enteric properties of Eudragit^®^ L100 and the absorption enhancing properties of TMC in a microsphere drug delivery system for oral administration of insulin. To our knowledge, this is the first microsphere delivery system comprised of Eudragit^®^ L100 and TMC that have been tested for enhanced macromolecular drug delivery. 

## 2. Results and Discussion

### 2.1. Synthesis of TMC

The degree of quaternization of the synthesized TMC polymer that was used in the microsphere formulations was calculated from its ^1^H-NMR spectrum to be 42%.

### 2.2. Scanning Electron Microscopy

**Figure 1:**
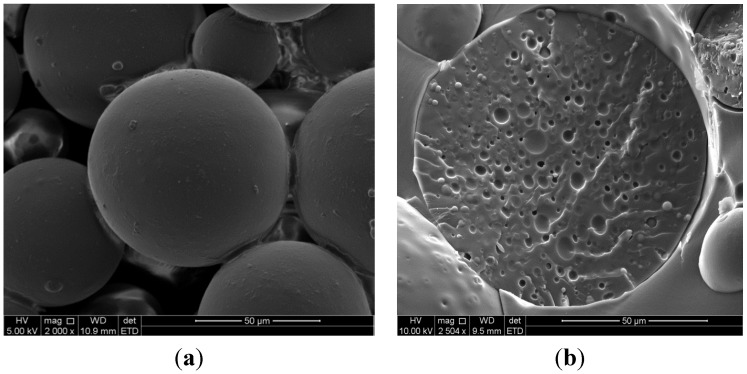
Scanning electron micrograph of a microsphere formulation consisting of Eudragit^®^ L100 (3.5% w/w), TMC (5% w/w) and insulin (2% w/w) showing (**a**) surface morphology (2000× magnification) and (**b**) internal structure (2500× magnification).

Electron microscopic characterisation of the eight microsphere formulations revealed that most formulations exhibited a spherical shape and smooth surface with a sponge-like internal structure as well as relatively good homogeneity in terms of size distribution. When higher concentrations of Eudragit^®^ L100 (7.5% w/w), TMC (10% w/w) and insulin (2% w/w) were incorporated, the shape of the spheres were irregular, probably due to the increased viscosity of the internal phase. Figure 1 shows an example of a scanning electron microscope (SEM) image of one of the microsphere formulations prepared from 3.5% w/w Eudragit^®^ L100, 5% w/w TMC and 2% w/w insulin.

### 2.3. Insulin and TMC Loading

Mean insulin loading of the eight microsphere formulations ranged from 27.9 ± 14.25 to 52.4 ± 2.72%, with an increase in insulin content when a higher concentration of insulin (2% w/w) was added to the formulation. Mean TMC loading of the microspheres (all formulations, A–H) ranged from 29.1 ± 3.3 to 37.7 ± 2.3%. Formulations with a higher Eudragit^®^ concentration (7.5% w/w) did not increase the insulin encapsulation efficiency, which can probably be attributed to the increased viscosity of the internal phase during microsphere preparation. Furthermore, the formulations prepared by using a higher concentration of TMC (10% w/w), did not result in the highest TMC loading. This could possibly be related to the processing conditions such as dispersion of TMC in the internal phase (*i.e.*, ethanol) and some un-incapsulated TMC particles may have been lost during isolation and washing of the microspheres. Based on the morphology and encapsulation results, two formulations (Formulation B and F as depicted in Table 1) were selected for further characterisation and evaluation. 

### 2.4. Particle Size

The mean particle size (based on volume, D[4.3]) of the two selected microsphere formulations was 157.3 ± 31.74 µm (Formulation B) and 135.7 ± 41.05 µm (Formulation F), respectively. Although the mean particle size of Formulation F was smaller than that of Formulation B, this difference was not statistically significant (*p* > 0.05). 

**Table 1:** Microsphere formulations A–H as defined by the fractional factorial design.

		TMC 5% w/w		TMC 10% w/w	
		Eudragit^®^ L100 (% w/w)		Eudragit^®^ L100 (% w/w)	
		7.5%	3.5%	7.5%	3.5%
**Insulin**	**2 (% w/w)**	Formula A	Formula B	Formula E	Formula F
	**1 (% w/w)**	Formula C	Formula D	Formula G	Formula H

### 2.5. Dissolution Behavior

**Figure 2:**
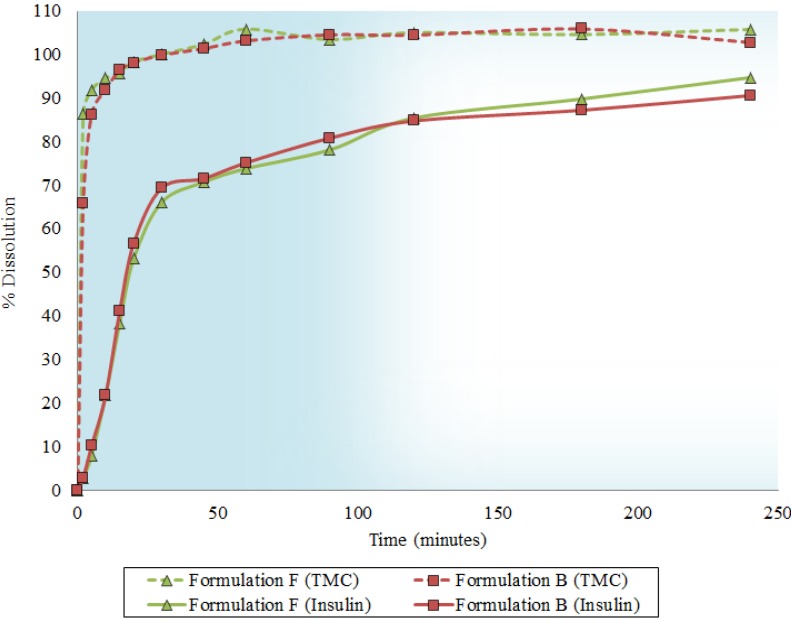
Insulin and TMC dissolution profiles of microsphere formulations B and F.

The dissolution profiles for insulin and TMC released from the microsphere formulations in a neutral environment (pH 7.4) are depicted in Figure 2. After correction factors were applied, the mean dissolution time (MDT) values for insulin were 34.5 ± 4.01 and 42.6 ± 9.06 min, while the MDT values for TMC were 1.2 ± 1.73 and 6.8 ± 6.42 min for Formulations B and F, respectively. A one-way analyses of variance (ANOVA) revealed no significant differences in the MDT of either insulin or TMC (*p* > 0.05) between the two formulations. However, the difference between the MDT values of insulin and TMC was significant (*p* < 0.05) for each formulation. The faster release of TMC may be beneficial, since TMC will be able to open the epithelial tight junctions prior to insulin release from the microspheres. The similarity factor (f_2_)–value obtained was higher than 50 (68.4 ± 8.18) and therefore the release profiles of Formulations B and F can indeed be considered similar. 

### 2.6. *In Vitro* Transport across Excised Intestinal Tissue

**Figure 3:**
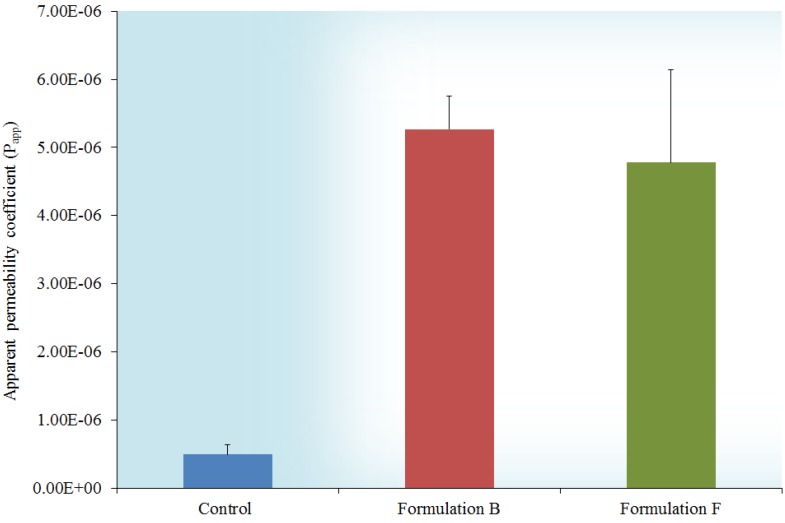
Apparent permeability coefficient (P_app_) values for insulin from two microsphere formulations and the control group.

**Figure 4:**
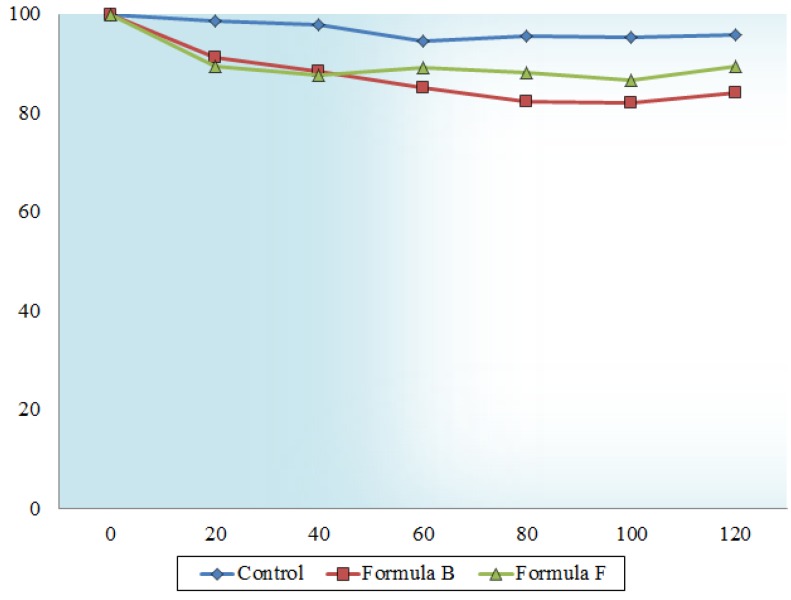
The transepithelial electrical resistance (TEER) of excised rat intestinal tissue treated with microsphere formulations B and F as well as control group plotted as a function of time.

The percentage cumulative transport of insulin over a period of 120 min across excised rat intestinal tissue was 8.3 ± 0.52% and 8.9 ± 2.26% of the initial insulin dose (t0) for Formulations B and F, respectively. Insulin transport from the TMC containing microsphere formulations when compared to each other showed no statistically significant difference (*p* > 0.05), however, it was statistically significantly higher (*p* < 0.05) compared to the transport of the control group (insulin only with cumulative transport of 0.7 ± 0.02%). The apparent permeability coefficient (P_app_) values calculated from the transport data are presented in Figure 3. The improved transport of insulin from the microsphere formulations compared to the control group can be attributed to the inclusion of TMC in the microspheres, which also reduced the transepithelial electrical resistance as shown in Figure 4.

It is evident from Figure 4 that the percentage reduction in TEER caused by the TMC containing microsphere formulations is more pronounced than that of the control group (insulin only). Formulation B showed a 15.9 ± 2.22% and Formulation F a 10.6 ± 5.29% reduction in TEER after a period of 120 min. In contrast, the control group lowered the TEER by only 4.11 ± 2.11%, which is significantly different from the microsphere formulations (*p* < 0.05). 

Microsphere formulations B and F released TMC at concentrations of 61.25 and 64.07 μg/mL, respectively, over a period of 240 min. These concentrations proved to be sufficient to cause substantial enhancement in the paracellular transport of the insulin across excised rat intestinal tissue. The transport enhancement ratios further emphasize the significant improvement in the transport of insulin released by the TMC containing microsphere formulations when compared to the control group. The transport enhancement rations of 10.67 and 9.68, respectively, indicate that the TMC containing microspheres are capable of increasing insulin transport across intestinal tissue in the order of 10-fold compared to the control group. 

A tiered approach is commonly used for scientific investigations on the pharmacokinetic aspects of drug delivery systems that usually commences with high throughput *in vitro* screening models, that have lower predictive power, followed by more predictive but lower throughput *in vivo* models. The *in vitro* investigation with the microspheres containing TMC was necessary to provide proof of concept. *In vivo* studies on the microsphere delivery system need to be done in future to confirm the clinical significance of the TMC containing microspheres in terms of enhanced drug delivery. 

## 3. Experimental

### 3.1. Materials

Eudragit^®^ L100 was a gift from Evonik Industries (Midrand, South Africa). The TMC was synthesized according to the method of Sieval *et al.* [20] from chitosan [97.6% deacetylated, 263,700 g/mole (Chitoclear™ Primex, Siglufjordur, Iceland)]. Recombinant human insulin, methyl iodide, ethanol (absolute), sorbitan mono-oleate (Span®80) and Krebs-Ringer bicarbonate (KRB) were purchased from Sigma–Aldrich (Kempton Park, South Africa). Liquid paraffin was purchased from Merck (Johannesburg, South Africa). Halothane was purchased from Safeline Pharmaceuticals (Roodepoort, South Africa). Sodium bicarbonate was purchased from Labchem (Edenvale, South Africa). Other reagents including *n*-hexane and polyoxyethylene sorbitan mono–oleate (Tween^®^ 80) were purchased from Associated Chemical Enterprises (Johannesburg, South Africa). All purchased chemicals were of analytical grade and used as received.

### 3.2. Synthesis of TMC

The TMC polymer was synthesized according to the two–step method of Sieval *et al.* [20], using similar reaction conditions, but also included an additional step. Briefly the method was as follows:

#### 3.2.1. First Reaction Step

A mixture of chitosan, sodium iodide, and an aqueous solution of sodium hydroxide in *N*-methyl-2-pyrrolidinone was stirred on a water bath at a temperature of 45 °C for 45 min. The methyl iodide was kept in the reaction by a Liebig condenser. The product was precipitated with ethanol and isolated by centrifugation.

#### 3.2.2. Second Reaction Step

The product obtained from step 1 was washed with ethanol and dissolved in *N*-methyl-2-pyrrolodinone. Sodium iodide, aqueous sodium hydroxide solution (15% w/v) and methyl iodide were then added. This mixture was stirred on a waterbath at a temperature of 60 °C for 30 min. The product was precipitated with ethanol and isolated by centrifugation.

#### 3.2.3. Additional Step

Before precipitation of the product at the end of reaction step 2, an additional volume of methyl iodide and sodium hydroxide pellets were added to the reaction mixture, which was then stirred for another 45 min at 60 °C, after which the product was precipitated with ethanol. In order to exchange the iodide ions with chloride ions, the product was dissolved in a 5% w/v aqueous solution of sodium chloride. The product was once again precipitated with ethanol and, to remove any residual sodium chloride, the product was dissolved in water and re–precipitated with ethanol to render the final product, which was washed twice with ethanol and diethyl ether and dried overnight under vacuum at 25 °C.

### 3.3. Microsphere Preparation

The emulsification/solvent evaporation technique is often selected as preparation method for microspheres owing to its versatility because drugs with a wide range of physicochemical properties can be successfully encapsulated ranging from small hydrophilic or hydrophobic molecules to proteins and genetic material [9,21,22]. 

Insulin-containing microspheres were prepared using a single water-in-oil (w/o) emulsification technique followed by solvent evaporation. The internal phase consisted of absolute ethanol (30 g) in which insulin (1–2% w/w), Eudragit^®^ L100 (3.5–7.5% w/w), TMC (5–10% w/w) and Tween^®^ 80 (1% w/w) were dissolved/dispersed based on the composition of the microsphere formulation to be prepared. Prior to microsphere preparation the synthesized TMC was placed into microcentrifuge tubes, followed by the addition of a carbide tungsten bead where after it was subjected to a vibration mill (Retsch GmbH & co. KG, Haan, Germany) for 5 min at 30 Hz to produce a fine powder. The TMC was then sieved (aperture 45 μm) in order to acquire a powder with uniform particle size, which was suitable for encapsulation in the microspheres from a dispersion. Firstly, Eudragit^®^ L100 (1.05 or 2.25 g, depending on the formulation) was dissolved in absolute ethanol (38 mL) followed by the dispersion of the TMC and insulin within the Eudragit^®^ solution, which was facilitated by a magnetic stirrer. Liquid paraffin (100 mL) containing Span^®^ 80 (1.5% v/v) was used as the continuous phase for the emulsion. A volume of 10 mL of the internal phase was added drop–wise at a rate of 20 rpm (±1 mL/min) to the continuous phase via an 18 gauge needle using a peristaltic pump (Watson Marlow Ltd., Falmouth, UK). The mixture was stirred constantly by means of a 4 cm diameter, ten-finned stirring disc at a stirring rate of 1,000 rpm with an overhead stirring apparatus (Heidolph, Schwabach, Germany) to assist the formation of the microspheres by accelerating solvent evaporation. After the addition of the internal phase to the continuous phase, the agitation of the emulsion was continued for approximately 15 h at 25 °C. The microspheres were separated by means of filtration and washed tree times with *n*-hexane to remove the residual liquid paraffin. The microspheres were stored at −8 °C until further evaluation.

A 2^3^ factorial design (Table 1) was used to investigate the combined influence of three variables namely Eudragit® L100 concentration, insulin concentration and TMC concentration on microsphere morphology as well as encapsulation efficiency. Based on the morphology and encapsulation efficiency, two microsphere formulations were selected from the initial eight formulations for further characterisation in terms of particle size distribution, dissolution behaviour and *in vitro* transport of insulin across excised intestinal tissue.

#### 3.3.1. Surface Morphology and Internal Structure

To investigate the morphology as well as the internal structure of the microsphere formulations, scanning electron microscope (SEM) images were captured with an FEI Quanta 250 FEG environmental scanning electron microscope (ESEM) with an integrated Oxford x-max EDS system (FEI Company, Eindhoven, Netherlands). Prior to capturing the images, all samples were coated under vacuum using an IB-2 Ion-Sputter-coater (Eiko Engineering, Ibaraki, Japan) with gold-palladium (70/30%), to minimize surface charge, followed by ESEM analysis at 5 or 10 kV. In order to visualize the internal structure, samples were embedded in resin (LR white, medium grade) and left for 16–24 h at 70 °C to set. After hardening of the resin occurred, the resin embedded samples were fractured using a blunt object to expose the internal structure of the microspheres. For each microsphere formulation, images of both the surface and internal structure were captured.

#### 3.3.2. Insulin Loading and TMC Loading

Based on the quantities of insulin weighed and included in the microsphere formulations, all microsphere formulations prepared in this study theoretically contained either an insulin concentration of 1% or 2% w/w. A mass of approximately 50 mg of each insulin containing microsphere formulation were transferred to volumetric flasks (25 mL) and made up to volume with PBS (pH 7.4). The samples were agitated by means of magnetic stirrers as well as sonification to ensure complete dissolution and analysed according to a validated HPLC method. Samples were analysed using an Agilent 1100 series high pressure liquid chromatograph (Agilent Technologies, Santa Clara, CA, USA) equipped with a gradient pump, autosampler and UV detector. All measurements were evaluated by Chemstation Rev. A.08.03 data acquisition and analysis software (Agilent Technologies, Tokyo, Japan). A Jupiter C18, 250 × 4.6 mm column, 5 μm spherical particles, with a 300 Å pore size, 13.3% carbon load, and endcapped (Phenomenex, Torrance, CA, USA) was used. The flow rate was set to 1.0 mL/min with an injection volume of 50 μL. The mobile phase consisted of acetonitrile (A) and 0.1% v/v orthophosphoric acid (B). The gradient conditions were 80% B, after 6 min, hold to 8 min, re-equilibrate at 20%, stop time 12 min. The wave length for UV detection was 210 nm. The retention time for insulin under these conditions was approximately 5.9 min.

The method employed to determine the TMC content was based on a colorimetric assay for the determination of chitosan in aqueous solutions [23], where a specific colour dye (*i.e.*, Cibacron Brilliant Red 3B-A) was added to the TMC containing solutions of which the absorbance of light at a wavelength of 572 nm was measured using a UV VIS SPECORD^®^ 200 spectrophotometer (Analytik Jena AG, Jena, Germany) equipped with a cartridge sipper system and WinASPECT PLUS version 4.1 spectroanalytical software.

#### 3.3.3. Particle Size Distribution

Particle size analysis of the selected formulations was conducted by means of laser diffraction with a Malvern Mastersizer 2000 instrument fitted with a Hydro 2000SM small volume dispersion unit (Malvern Instruments, Malvern, UK). Deionized water was used as dispersion medium at a stirring rate of 1,500 rpm. Samples were dispersed in 5 mL of deionized water prior to addition to the small volume dispersion unit and each measurement consisted of 12,000 sweeps.

#### 3.3.4. Dissolution Studies

Dissolution studies were conducted to investigate the release characteristics of insulin as well as TMC from the microsphere formulations using a rotating bottle apparatus. The samples were rotated at a speed of 25 rpm and a volume of 2 mL from each sample were withdrawn at intervals of 2, 5, 10, 15, 20, 30, 45, 60, 90, 120, 180 and 240 min following the start of the experiment, using a filter unit fitted with a glass fibre pre-filter (Sartorius Stedim Biotech GmbH, Goettingen, Germany). The volume withdrawn was replaced with an equal volume of blank PBS (pH 7.4) at a temperature of 37 °C to keep the dissolution volume in the glass bottles constant. Initially, samples (100 mg) of each microsphere formulation were transferred to amber glass bottles (50 mL) containing 40 mL of PBS (pH 7.4) at a temperature of 37 °C. The glass bottles were sealed with Parafilm^®^ as well as a screw cap, where after the bottles were secured on the rotating bar. After withdrawal of the sample at 240 min, the bottles were sonificated for 15 min to determine the total insulin content. From the volume withdrawn the amount of TMC as well as insulin released were determined. In small volume dissolution studies, replenishment of the sampled volume with fresh dissolution medium can lead to dilution of the system, resulting in a negative slope on the dissolution curve as the system approaches equilibrium solubility. Early in the dissolution study, while the drug solubility is larger than its concentration in the dissolution medium (*Cs* >>*C*), the dilution is negligible because the system is operating under sink conditions. However, as the solute concentration in the bulk solution approaches that of *Cs* in the diffusion layer, the effect of the dilution becomes more apparent. Since the static diffusion layer is nearly saturated, any dilution of the bulk solution can be expressed as a fraction relative to the static diffusion layer’s concentration. If the last data point on the dissolution curve displaying a positive slope is designated as the concentration where the system approximates equilibrium solubility, *Cs_approx_*, a correction factor, can be calculated for each diluted concentration using the following equation:

(1) $$\frac{\text{dM}}{\text{dt}}=\frac{\text{DS}}{\text{h}}\left(\text{Cs}-\text{C}\right)$$

where M is the mass of solute dissolved in time t, dM/dt is the dissolution rate, D is the diffusion constant, S is the surface area of the exposed solid, h is the thickness of the diffusion layer, Cs is the solubility of the solid and C is the concentration of the solute in the bulk solution at time t [24]. 

According to mass transfer theory, a static (or stagnant) liquid layer of thickness h exists on the surface of a dissolving solid, with solute concentrations ranging from C to Cs. As long as Cs >>C, the system can be represented by sink conditions, however, as C approaches Cs the dissolution rate will decrease until equilibrium solubility is established. This corrected concentration represents the undiluted concentration.

In order to determine the extent of drug dissolution as well as the mean time taken for the drug to dissolve under *in vitro* dissolution conditions, the mean dissolution time (MDT) was calculated. Additionally, f_2_ was calculated in order to compare the difference between the percentage drug dissolved per unit time for each formulation and the reference [25]. 

#### 3.3.5. *In Vitro* Transport Studies

Adult male FSR rats (400–450 g) were obtained from the Animal Research Centre (North West University) and the study was approved by the University’s ethics committee under protocol number NWU-0018-09-A5. All animals used had free access to food and water prior to sacrifice and were selected at random. For preparation of tissue segments, rats were anaesthetized with halothane and an incision was made in the thorax, to ensure death. An incision was then made in the abdomen to expose the small intestine and a 20–30 cm piece of proximal jejunum (10 cm from the pylorus sphincter of the stomach) was excised and washed thoroughly with ice–cold KRB buffer (±10 °C). Thereafter, the intestinal tissue was pulled over a glass rod. The excised tissue was then gently scoured along the mesenteric border by blunt dissection in order to remove the serosa and muscle layer by using the index finger along the length of the jejunum. Throughout the procedure, the intestinal tissue was kept moist with cold KRB buffer and was kept in an ice bath. The excised jejunum was then cut open along the mesenteric border with a scalpel blade and washed off the glass rod with KRB onto a strip of filter paper to form a flat epithelial sheet. The excised tissue and filter paper was then cut simultaneously into pieces of approximately 3 cm in length. Segments containing Peyers’ patches were identified visually and avoided as their morphology and thickness may cause greater variation in the rates of transport between compartments. The segments were then carefully mounted onto the pins of the preheated (37 °C) half-cells. The filter paper was used to minimise direct contact so as to maintain tissue viability, where after it was carefully removed and the matching half-cells were clamped together using a metal ring. Assembled cells were placed into the heating block (preheated to 37 °C) and 7 mL of preheated (37 °C) KRB buffer were added to each compartment. Circulation as well as oxygenation of buffer was maintained by a gas lift of 95% O_2_: 5% CO_2_ at a flow rate of approximately 15 mL/min. After the cells were placed in the heating block with their gas supply, it was left for 15 min for the tissue to adapt to the environment and reach a state of equilibrium. The entire procedure from the first incision till the system reached a state of equilibrium was performed within 45 min.

The transport of insulin across excised rat jejunum was determined in the apical to basolateral (AP-BL) direction over a period of 120 min. The KRB buffer in the chambers was completely removed from both sides using an Integra vacusafe aspiration system (Labotec, Johannesburg, South Africa) and replaced by a volume of 7 mL of the microsphere suspensions in KRB buffer (pH 7.4) at an insulin concentration of 170 μg/mL on the apical side and 7 mL KRB buffer on the basolateral side. The microsphere dispersion remaining after the transport study was sonicated and used as 100% concentration (time 0 or t_0_) to calculate the percentage of the dose transported across the excised tissue at each time point. Furthermore, samples of 200 μL were withdrawn every 20 min from the basolateral side from all six chambers over a period of 120 min. The TEER was measured along with each sample withdrawal using a Millicell^®^ ERS-2 epithelial volt-ohm meter (Millipore Corporation, Darmstadt, Germany) in order to evaluate the integrity of the excised tissue as well as the effect of the TMC (*i.e.*, opening of tight junctions). The samples withdrawn from the basolateral side were immediately replaced with an equal volume of KRB buffer (preheated, 37 °C). A control group was included in the experimental design to determine the transport of insulin without the microparticulate delivery system containing TMC as permeation enhancer. The control experiment was used to indicate that the effects seen with the TMC and polymer delivery system were caused by their presence and not by chance interferences or external factors. 

Transport samples were analysed by means of high performance liquid chromatography (HPLC) to determine the percentage transport. The concentration of the transport samples was corrected for dilution and expressed as cumulative transport (% of initial dose). To further compare the absorption enhancement obtained, apparent permeability coefficient values (P_app_) for insulin as well as transport enhancement ratios were calculated. The P_app_ is an index widely used as part of a general screening process to study drug absorption and is defined as the initial flux of a compound through the membrane, normalized by membrane surface area and donor concentration [26].

## 4. Conclusions

Insulin as well as TMC was successfully incorporated into microsphere formulations prepared by the emulsification solvent evaporation method. TMC was released at a faster rate than insulin from the microspheres. This may be beneficial, since TMC will be able to open the tight junctions prior to insulin release, which may minimize insulin lost through intestinal transit. The increase in insulin transport across excised rat intestinal tissue by the microsphere formulations compared to that of the control group (*i.e.*, insulin alone) correlated well with the decrease in TEER, indicating the opening of the tight junctions to allow for paracellular transport of insulin. Thus, the opening of the tight junctions by TMC containing microspheres represents a potential approach to increase the paracellular absorption of peptide and protein drugs such as insulin for effective oral delivery. 

## References

[1] Pauletti G.M., Gangwar S., Knipp G.T., Nerurkar M.M., Okumu F.W., Tamura K., Siahaan T.J., Borchardt R.T. (1996). Structural requirements for intestinal absorption of peptide drugs. J. Controlled Release.

[2] Hamman J.H., Enslin G.M., Kotzé A.F. (2005). Oral delivery of peptide drugs: barriers and developments. Biodrugs.

[3] Morishita M., Peppas N.A. (2006). Is the oral route possible for peptide and protein drug delivery. Drug Discovery Today.

[4] Daugherty A.L., Mrsny R.J. (1999). Transcellular uptake mechanisms of the intestinal epithelial barrier: part one. Pharm. Sci. Technol. Today.

[5] Park K., Kwon I.C., Park P. (2010). Oral protein delivery: Current status and future prospect. React. Funct. Polym..

[6] Ziv E., Lior O., Kidron M. (1987). Absorption of protein via the intestinal wall. Biochem. Pharmacol..

[7] Hosny E.A., Al-Shora H., Elmazar M.M.A. (2002). Oral delivery of insulin from enteric-coated capsules containing sodium salicylate: Effect on relative hypoglycemia of diabetic beagle dogs. Int. J. Pharm..

[8] Nilkumhang S., Basit A.W. (2009). The robustness and flexibility of an emulsion solvent evaporation method to prepare pH-responsive microparticles. Int. J. Pharm..

[9] Singh M.N., Hemant K.S.Y., Ram M., Shivakumar H.G. (2010). Microencapsulation: a promising technique for controlled drug delivery. Res. Pharm. Sci..

[10] Salamat-Miller N., Johnston T.P. (2005). Current strategies used to enhance the paracellular transport of therapeutic polypeptides across the intestinal epithelium. Int. J. Pharm..

[11] Van Der Merwe S.M., Verhoef J.C., Verheijden J.H.M., Kotzé A.F., Junginger H.E. (2004). Trimethylated chitosan as polymeric absorption enhancer for improved peroral delivery of peptide drugs. Eur. J. Pharm. Biopharm..

[12] Thanou M., Verhoef J.C., Junginger H.E. (2001). Oral drug absorption enhancement by chitosan and its derivatives. Adv. Drug Deliver. Rev..

[13] Thanou M., Verhoef J.C., Junginger H.E. (2001). Chitosan and its derivatives as intestinal absorption enhancers. Adv. Drug Deliver. Rev..

[14] Kotzé A.F., Lueßen H.L., De Boer A.G., Verhoef J.C., Junginger H.E. (1998). Chitosan for enhanced intestinal permeability: prospects for derivatives soluble in neutral and basic environments. Eur. J. Pharm. Sci..

[15] Kotzé A.F., Lueßen H.L., De Leeuw B.J., De Boer A.G., Verhoef J.C., Junginger H.E. (1998). Comparison of the effect of different chitosan salts and *N*-trimethyl chitosan chloride in the permeability of intestinal epithelial cells (Caco-2). J. Control. Release.

[16] Thanou M.M., Verhoef J.C., Romeijn S.G., Nagelkerke J.F., Merkus F.W.H.M., Junginger H.E. (1999). Effects of *N*-trimethyl chitosan chloride, a novel absorption enhancer, on Caco-2 intestinal epithelia and the ciliary beat frequency of chicken embryo trachea. Int. J. Pharm..

[17] Snyman D., Hamman J.H., Kotzé A.F. (2003). Evaluation of the mucoadhesive properties of *N*-trimethyl chitosan chloride. Drug Dev. Ind. Pharm..

[18] Kotzé A.F., Thanou M.M., Lueen H.L., de Leeuw B.J., de Boer A.G., Verhoef J.C., Junginger H.E. (1999). Enhancement of paracellular drug transport with highly quaternized *N*-trimethyl chitosan chloride on the permeability of intestinal epithelial cells (Caco-2). J. Pharm. Sci..

[19] Hamman J.H., Schultz C.M., Kotzé A.F. (2003). *N*-trimethyl chitosan chloride: optimum degree of quaternization for drug absorption enhancement across epithelial cells. Drug Dev. Ind. Pharm..

[20] Sieval A.B., Thanou M., Kotzé A.F., Verhoef J.C., Brussee J., Junginger H.E. (1998). Preparation and NMR characterization of highly substituted *N*-trimethyl chitosan chloride. Carbohyd. Polym..

[21] Watts P.J., Davies M.C., Mella C.D. (1990). Microencapsulation using emulsification/solvent evaporation: An overview of techniques and applications. Crit. Rev. Ther. Drug Carrier Syst..

[22] Rosca I.D., Watari F., Uo M. (2004). Microparticle formation and its mechanism in single and double emulsion solvent evaporation. J. Controlled Release.

[23] Muzzarelli R.A.A. (1998). Colorimetric determination of chitosan. Anal. Biochem..

[24] Martin A.N., Bustamante P., Chun A.H.C. (1993). Physical Pharmacy: Physical Chemical Principles in the Pharmaceutical Sciences.

[25] Moore J.W., Flanner H.H. (1996). Mathematical comparison of dissolution profiles. Pharm. Technol..

[26] Palumbo P., Picchini U., Beck B., Van Gelder J., Delbar N., Degaetano A. (2008). A general approach to the apparent permeability index. J. Pharmacokinet. Phar..

